# Integrated, Not Isolated: Defining Typological Proximity in an Integrated Multilingual Architecture

**DOI:** 10.3389/fpsyg.2017.02212

**Published:** 2018-01-04

**Authors:** Michael T. Putnam, Matthew Carlson, David Reitter

**Affiliations:** Center for Language Science, The Pennsylvania State University, Pennsylvania, PA, United States

**Keywords:** typological proximity, bilingualism, computational modeling, parallel architectures, vector space models

## Abstract

On the surface, bi- and multilingualism would seem to be an ideal context for exploring questions of typological proximity. The obvious intuition is that the more closely related two languages are, the easier it should be to implement the two languages in one mind. This is the starting point adopted here, but we immediately run into the difficulty that the overwhelming majority of cognitive, computational, and linguistic research on bi- and multilingualism exhibits a monolingual bias (i.e., where monolingual grammars are used as the standard of comparison for outputs from bilingual grammars). The primary questions so far have focused on how bilinguals balance and switch between their two languages, but our perspective on typology leads us to consider the nature of bi- and multi-lingual systems as a whole. Following an initial proposal from Hsin (2014), we conjecture that bilingual grammars are neither isolated, nor (completely) conjoined with one another in the bilingual mind, but rather exist as integrated source grammars that are further mitigated by a common, combined grammar (Cook, 2016; Goldrick et al., 2016a,b; Putnam and Klosinski, 2017). Here we conceive such a combined grammar in a parallel, distributed, and gradient architecture implemented in a shared vector-space model that employs compression through routinization and dimensionality reduction. We discuss the emergence of such representations and their function in the minds of bilinguals. This architecture aims to be consistent with empirical results on bilingual cognition and memory representations in computational cognitive architectures.

## Introduction

The concept of typological proximity/distance has long been a useful one in language science, but despite its intuitiveness on many levels, it remains maddeningly difficult to measure in any large-scale sense. Part of the problem, we argue, is that its development and consequences at the diachronic vs. the synchronic levels have not yet been sufficiently articulated. Diachronically, a great deal of attention has long been paid to the evolution of grammars, from sound change to morphosyntax (Fedzechkina et al., 2012), and historical linguistics has made enormous contributions to our understanding of language, and provides (among other things) ways of understanding typological distance as instantiated in language phylogeny. However, in most cases, our only evidence of phylogenetic relationships are the synchronic correspondences among putatively related languages, meaning that diachronic measures of typological distance are generally based on synchronic correspondences between languages. Frequently, lexical overlap forms the basis for these classifications, but where this fails, as in Papuan and Oceanic languages, researchers have attempted to make classifications on the basis of shared grammatical features (Dunn et al., 2005). The fundamental problem here is that the researcher must decide what grammatical features are to be used.

Moreover, typological relatedness in the synchronic sense plays an important role in understanding phenomena associated with bilingualism, including second language acquisition, language transfer, attrition, and code-switching, and in this domain, both genetic relationships among languages and also proximity due to convergent evolution are important. What is needed, therefore, is a general concept of typological proximity that can serve as a foundation for a metric that is independent of the source of that proximity, and one that is not based on arbitrary decisions made by the researcher (see e.g., also similar criticisms directed at the generative notion of “parameter” by Newmeyer, 2004, 2005). Specifically, to the extent that any human language can be situated within a common space of possible languages implies that typological distance is measurable synchronically as well, regardless of its source.

This more synchronic conceptualization of typological proximity has played a larger role in second language acquisition research and related sub-disciplines, both explicitly (in various instantiations of the idea of contrastive analysis, going back at least to Lado, 1957), and implicitly (Recchia et al., 2010). This research, too, has tended to focus on specific shared features or families of features, with the general intuition that second language learning proceeds more easily where there is overlap, and that contrast presents more challenges (though partial overlap may present the greatest challenges, e.g., Flege, 2007). The impact of correspondence and contrast between two grammars in second language acquisition is, however, just a specific instantiation of much more general questions about how two or more grammars are instantiated in the multilingual mind, questions that have garnered increasing attention in recent years (Grosjean, 1989; Cook, 1992, 1995; Kecskes, 1998; Roeper, 1999; Kecskes and Papp, 2000; Hall et al., 2006; Braunmüller, 2009; Amaral and Roeper, 2014; Grohmann, 2014; Cook and Wei, 2016, and references therein).

The major intuition in this research is that grammars are not instantiated side-by-side in the multilingual mind, but they are integrated into a single, compound system. What we argue here is that this integration provides a useful way of conceptualizing, and even measuring, the typological proximity of language pairs. It can thus fill the gap in understanding the direct, synchronic relatedness of two grammars, independent of the diachronic histories that brought them to that point. Moreover, via a second important intuition, that language change grows out of synchronic variation, and that the representation of variation can be thought of as a form of multilingualism, this synchronic view of typological proximity can be integrated with the diachronic one, leading to a more comprehensive view of how this proximity arises, and how it shapes the competence and usage of individual language users. Of course, the idea that multilingualism contributes to language change (thus contributing to linguistic relatedness) has long been acknowledged, particularly in the subdiscipline of contact linguistics (Thomason and Kaufman, 1992 is but one substantial example). But the original focus there was on whether and how specific structures at various levels of linguistic description can pass from one language into another, whereas our proposal is much more comprehensive: that by conceptualizing the language knowledge of a multilingual (or a monolingual, counting variation) as a single grammatical system, and comparing the result to a coordinate system, where both languages are represented side by side, but independently, we can gain vital insight into the notion of typological distance.

Here we introduce the core aspects of an algorithm which can measure typological proximity/distance between languages. Importantly, our primary focus here is on modeling typological proximity in the bilingual mind, which requires the inclusion of a common, combined grammar that we will discuss below. The key to all of this, of course, is to approach a well-developed understanding of what it means for two languages to be “integrated” in one mind. Here we discuss the fundamental ontology of an integrated grammar and how typological similarities and differences can be accounted for in a clear and systematic way.

## A holistic view - integrated grammars

Research in cognitive neuroscience over the past three decades has provided a cascade of evidence that both languages are, to various degrees, simultaneously active in the mind of bilinguals (e.g., Hartsuiker et al., 2004; Deuchar, 2005; Pickering and Ferreira, 2008; Coppock, 2010; Hsin et al., 2013; Kroll and Gollan, 2014; Melinger et al., 2014; Starreveld et al., 2014; de Groot, 2016). Such research has gathered steam since initial proposals from pioneers such as Grosjean (1989) who advanced a holistic view of language, language development, and language use in bilinguals. The impact of this body of research issues significant challenges to research on modeling techniques that seek to better understand the emergence of grammar in individuals, and to an extent, our species. These findings have a profound impact on the (generative) models that we impose on the grammatical competence of multilinguals, as suggested by de Bot (2004), Hall et al. (2006), and Roeper (1999). Cook (2016, section 1.4) lists three primary premises regarding the role of the multi-competent native speaker:

**Premise 1:** Multi-competence concerns the total system for all languages (L1, L2, Ln) in a single mind and their inter-relationships.
**Premise 2:** Multi-competence does not depend on monolingual native speakers.
**Premise 3:** Multi-competence affects the whole mind, i.e., all language and cognitive systems, rather than language alone.

In the remainder of this article, we will focus primarily on the first two of Cook's premises, while acknowledging that we agree with the third and final premise, but will not address it directly due to space constraints (see e.g., Jarvis and Pavlenko, 2008). The initial charge to treat bilingual grammars on par with monolingual grammars, i.e., as natural/authentic grammars, led to proposals such as the *Null Hypothesis* (Mahootian, 1993), which banned the postulation of constraints and representations that were strictly unique to bilingual grammars. In spite of these advances, work on bilingualism—especially research on the language of late bilinguals—tends to be “deficit”-oriented (Ortega, 2014), i.e., with the focus on differences between target outputs being the result of some sort of competence or production deficit of one of the source grammars.

This perspective can challenge the validity of treating both grammars in the mind of an individual as “natural languages.” In our integrated perspective, we adopt Ortega's (2016, pp. 50–51) proposal—following initial proposals by Cook (2012, 2016)—that “linguistic competencies and indeed language itself are dynamic and they change at multiple time scales, including over the lifespan, as the function of actual use (Beckner et al., 2009; de Bot et al., 2013).” Of equal importance, the influence of one source grammar upon another need not be unidirectional; much research (e.g., Kecskes and Papp, 2000; Cook, 2003; Flege, 2007; and others) provides evidence that such influence is bidirectional. Finally, again as pointed out by Ortega (2016, p. 51), “language is part of cognition and, as such, cognition and language influence and affect each other (Langacker, 2008; Pulvermüller, 2013; Bylund and Athanasopoulos, 2014).” Here we sketch out the key underpinnings of an integrated cognitive architecture, while remaining true to Mahootian's (1993) *Null Hypothesis* that bans the inclusion of features, operations, and constraints that are unique to bilingual grammars.

In the remainder of this article we take a bold step forward in attempting to unite these observations about the nature of multi-competence with current cognitive models and linguistic theorizing. Building upon an initial proposal by Hsin (2014), which we will explicate in more detail in the next section, we call for an integrated view of grammatical competence in the bilingual mind. To be clear, our adoption of an integrated grammar should not be confused with previous attempts in the generative tradition to come to terms with the simultaneous acquisition of grammar in bilingual children. In this literature, there are two dominant positions; the fused or unified development hypothesis (Volterra and Taeschner, 1978; Taeschner, 1983) and the isolation hypothesis (Meisel, 1990). According to the former, the initial state would consist of a unified, or “common” grammar, which, over time, bilingual children would begin to gradually differentiate into (largely) separate source grammars. Hybrid representations found in code-mixing served as the initial empirical support for this hypothesis^1^. In contrast, the latter hypothesis also draws on code-mixing data, but builds upon the observation that although there exists a high degree of lexical items from both source grammars in such hybrid representations, the amount of cross-linguistic influence from both syntactic systems is relatively scarce. Meisel's (1990) proposal that simultaneously developing grammars remain (mostly) isolated from one another thus contrasts with the former proposal. Hsin (2014, pp. 6–7) introduces a third option, which she calls the integration hypothesis which “embodies an account in which bilingual children indeed begin with the same basic endowments […] as monolingual children, and […] where the two languages diverge with respect to a particular syntactic rule, the grammar responds by duplicating, or splitting, the constraint that is not satisfied for both languages.” We demonstrate below that these degrees of freedom are necessary in coming closer to an accurate, working model of multilingual competence.

Remaining consistent with the general theme of this Frontiers volume, we then explore how a model that adopts some version of the integration hypothesis can accurately model the typological proximity (and, conversely, distance) between entire linguistic systems. As we discuss below, what is needed is a model that extends beyond the traditional notion of (innate) parameters (a concept that Cook, 1991, already began to adjust in his initial proposal of multicompetence), as suggested in the ongoing research carried out in the Principles and Parameters model (P&P, Chomsky, 1982) and beyond. Recent theorizing has sought to eliminate the reliance on such parameters for a number of reasons, opting instead for “realization options” (Boeckx, 2016, p. 90; also see Roeper, 2016 for similar ideas)^2^. To briefly clarify this point, operations in the Narrow Faculty of Language (Hauser et al., 2002) are reduced significantly to notions of Merge, (possibly) recursion, and another subset of locally-defined operations (such as Agree and c-command) (see e.g., Chomsky et al., 2017 for a detailed overview of the current state of this research program). The generative component of this model is relatively unrestricted and unconstrained when compared with previous instantiations of the P&P-framework, where elements that were previously interpreted as catalysts for syntactic operations (e.g., Case, wh-movement, etc.), now become realization options external to the computational systems (i.e., at the hands of “external” interfaces). Under such assumptions, traditional “parameters” exist outside of the Narrow Faculty of Language (Hauser et al., 2002) and cross-linguistic variation is thus relegated to “third factor” considerations (Chomsky, 2005). We welcome this development for a number of reasons, most notably, because it presents a platform to unite theorizing traditionally thought to be unique to generative inquiry to a larger body of cognitive science. In the third section of this report, we discuss how these recent developments can be integrated into an emergent model of language acquisition, such as that proposed and developed by MacWhinney (2005, 2008).

In the sections that follow, we flesh out our proposal of the general cognitive architecture that underlies a multi-competence language faculty. The fourth section of our report lays out the conceptual motivation and foundation for our model, while the fifth and final section advances a novel sketch of the core desiderata that would be deployed in such a system.

## Dynamic integration

If we are to move beyond the monolingual biases discussed by Cook (2012, 2016) and Ortega (2016) in an attempt to develop a cognitive architecture, we need to approach such an endeavor with our own set of axioms:

**Axiom 1:** Mental representations and their sub-components are lossy and gradient by nature. The reliability and stability of representations can be affected by myriad factors such as proficiency, working memory constraints, and activation/usage^3^.
**Axiom 2:** These mental representations only exhibit temporary “resting periods” or, *attractor states*, although these states may often be extremely stable and long-lasting.
**Axiom 3:** Parametric variation is no longer (primarily) tied to parameters licensed in a narrow computational faculty (i.e., the narrow syntax), and are now external from this core architecture.

Our first axiom shares many similarities with Cook's Premise 1 to the extent that both assume the competence of bi/multilinguals to be an amalgamation of all contributing source grammars. The very existence of mental representations is of paramount importance in understanding and modeling cognition, as explained by Kühn and Cruse (2005, pp. 344–345):

*By means of these representations, the behavior can be uncoupled from direct environmental control. This enables the organism, for example, to respond to features of the world that are not directly present, to use past experiences, to shape present behavior, to plan ahead, to manipulate the content, etc. (Cruse*, *2003**). All of these instances characterize a special feature of language called ‘displacement’ (Hockett*, *1960**). Therefore we conclude that these mental representations form an essential prerequisite to explaining how organisms can behavior in a cognitive way*.

Importantly, in becoming a unified linguistic system^4^, this amalgamation must cope with varying degrees and concentrations of correspondence between the source systems. This is what leads us to Axiom 1, where features that distinguish similar patterns across two source systems may play a lesser or greater role in representation and processing, depending on the usefulness of the commonalities.

The second Axiom grows out of an important fact about bilingualism, which is that usage patterns change over time. People may become bilingual at different times as well as to different degrees, and the balance of usage may shift toward or away from any given (source) language. Bilingualism thus demands a notion of grammar and mental representation that is generally stable, but underlyingly dynamic, much more clearly than monolingualism, where the underlying dynamism is much less apparent.

Lastly, concerning Axiom 3, the responsibility of the grammar is to generate environments where this unified grammar network can establish instances of congruency. This occurs in monolingual grammars, e.g., in the piecemeal acquisition of structures whose generality is not grasped by the child until later (Yang, 2002). Extending this reasoning to bilingual systems, the relatedness of structures in each language must be reflected in the representational resources at the core of the multilingual system (i.e., bilinguals' knowledge of overlap in their systems does not merely stem from metalinguistic reflection). This in turn implies a novel view of typological relatedness as the degree to which congruency can be established across languages in a combined system.

We view the establishing of congruency and the architecture that it takes place in to be dynamic and emergent, but importantly, this does not eliminate the need for formal theorizing. On the contrary, as we argue here, this view of the cognitive architecture underlying the language faculty strongly supports the integration of competing linguistic information from multiple source grammars at designated points in the grammatical structure. In summary, the consequence of these axioms, and the integrated view we take here, for typological distance is that the kinds, amount, and degree of overlap or correspondence between source grammars is expected to strongly shape the way that the integration plays out, and that a global understanding of typological relatedness falls out from the way bilinguals use that overlap to build an integrated, multicompetent language system.

At this juncture, it will be useful to visit some of the data that motivates this view, and certain research topics where this kind of reasoning is developing, which in turn will provide guidance for where to seek further evidence and test the predictions that will grow out of a more formalized approach to the notions of overlap and equivalence in these aforementioned ways. As examples, we discuss evidence from the literature on code-switching, cross-linguistic structural priming, typological/genetic relationships, bilingual speech, and L2 acquisition.

An obvious domain where these notions of overlap and equivalence take center stage is in code-switching. Although code-switched utterances are frequently analyzable as relying on one source grammar, leading to a strong role in code-switching research for the idea that one grammar is in play at a time (e.g., Myers-Scotton, 2001), this is not always the case, and it has proven difficult—if not impossible—to derive absolute rules and strict constraints to account for these data. To address this point, Goldrick et al. (2016a,b) employ a probabilistic grammar model known as Gradient Symbolic Computation (GSC; Smolensky et al., 2014) that shares many affinities with other earlier versions of Harmonic Grammar (HG; Legendre et al., 1990; Smolensky and Legendre, 2006). To account for the fact that both grammars are active to various degrees in the mind of a bilingual, Goldrick et al. propose a calculation that determines the strength of each contributing source grammar as well as a “common” grammar (which is consistent with Cook's Premise 1 discussed above). These values then interact with higher-level cognitive symbols (i.e., violable constraints), which evaluate input-output candidate representations to determine a Harmony profile for each pair. Importantly, the yielded Harmony value of each input-output pair contributes to the probability of occurrence of each representation relative to one another. In other words, every representation that has a non-zero probability of occurrence is essential for computing the probability of a particular form in relation to all possible forms. Hybrid representations containing various lexical and grammatical elements may differ to the extent that they include elements from source grammars (although it is a ubiquitous assumption that one of the source grammars functions as the matrix/dominant language in switches). Putnam and Klosinski (2017) extend the initial work of Goldrick et al. by investigating two different types of code-switches that vary with respect to the degree that both grammars contribute to hybrid representation. They make the distinction between mixes and blends (see also Chan, 2008, 2009), illustrated below in (1) and (2):

(1) Mix: Welsh-English Determiner Phrase (Parafita Couto and Gullberg, 2016, p. 855):
y       Belgian loaf
det^W^ Belgian^E^ loaf^E^
‘the Belgian loaf’
(2) Blend: Verb + Adverb English-Japanese (Nishimura, 1986, p. 139)
We bought about two pound gurai kattekita no.
we bought about two pounds about bought tag
‘We bought about two pounds.’

Mixes are hybrid representations that consist of lexical items from both/multiple source grammars but appear to only follow one particular source grammar for structural purposes. In the mix-example in (1) above, the determiner phrase (DP) contains a mixture of lexical elements from both source grammars, but, crucially, only the English order of Det(erminer)-Adj(ective)-N(oun) appears (Welsh: Det-N-Adj). In contrast, blends are representations where elements of both source grammars appear in the representation [i.e., the Verb + Adverb – Adverb + Verb orderings in the English-Japanese blend in (2)]^5^. The work of Goldrick et al. (2016a,b) and Putnam and Klosinski (2017) provide a working metric to determine the activation and gradient nature of bi/multilingual representations. Importantly, this approach is consistent with an integrationist approach to the bilingual cognitive architecture as well as the Null Hypothesis (Mahootian, 1993) and Cook's (2016) Premise 1 (listed above) for the following reasons: First, at no point do they assume that both grammars are truly either fused/united or isolated from one another. The activation and strength of representation (which could also be construed to be an analog for proficiency) of each grammar contributes to determine the value of a shared “common” grammar at a given point in time. It is crucial to reiterate that the value of this “common” grammar can be altered at a given time and over the course of a longer period of time due to a variety of mitigating factors; e.g., priming effects, lack of activation/usage, etc. Second, at no point does this model require features, constraints, or axioms that are solely unique to bi/multilingual cognition and its representations.

Additional evidence that forces us to revisit and better define the notions of overlap and equivalence comes from psycholinguistic data on syntactic priming effects (e.g., Bock, 1986; Branigan and Pickering, 1998; Bernolet et al., 2007; Schoonbaert et al., 2007). This work can provide valuable constraints pertaining to the nature of grammatical representations: how categorical they are, what is their granularity, and what are the mechanisms for general implicit (non-declarative) and procedural memories shared with those storing lexico-syntactic information. A model formulating syntactic storage within a hybrid symbolic/sub-symbolic cognitive architecture (Reitter et al., 2011) has seen several empirical predictions borne out (e.g., Kaschak et al., 2011; Segaert et al., 2016), including that such priming is modulated by the long-term activation (frequency) of syntactic information in the same way in L1 and in L2 speakers (Kaan and Chun, 2017). This lends credence to joint representational mechanisms (i.e., hybrid symbolic/subsymbolic representations), regardless of age of acquisition. As a case in point, Jacob et al. (2016) conducted two cross-linguistic priming experiments with L1 German-L2 English speakers where they investigated both the role of constituent order and level of embedding in cross-linguistic structural priming. The results of these experiments showed significant priming effects in connection with two factors: (i) whenever both languages shared the same constituent order, and (ii) when both languages were identical with regard to level of embedding.

This kind of cross-linguistic effect also extends to morphosyntactic features, such as gender. In a visual world study conducted by Morales et al. (2016), Italian-Spanish bilinguals and Spanish monolinguals listened to sentences in Spanish while viewing an array of pictures, one of which was the target in the sentence. The objects in the experiment depicted elements that either shared the same gender in both languages, or were mismatched with respect to gender assignment. Bilinguals looked less at the target object when its Italian gender mismatched its Spanish gender, suggesting that, to some degree, both gender features were active as the sentence was interpreted, even though the experiment was exclusively in Spanish. Additional studies by Paolieri et al. (2010a,b) offer further evidence of morphosyntactic interactions in bilingual grammars, and research by Malt et al. (2015) demonstrates similar phenomena in the domain of semantics.

In the realm of bilingual speech, the mapping of phonetic space to speech sounds has long been an active area of inquiry (e.g., Flege, 2003; Best and Tyler, 2007; Gonzalez and Lotto, 2013), but recent evidence suggests integration of phonological systems at more abstract levels. Carlson et al. (2016) tested fluent, early Spanish-English bilinguals on a perceptual illusion related to Spanish phonotactics. Specifically, word-initial /s/-consonant clusters are prohibited in Spanish, and are obligatorily repaired by prepending an [e]. Presented with acoustic stimuli beginning with the illicit clusters, Spanish speakers tend to perceive an illusory [e], but this effect was lessened in Spanish-English bilinguals, and more so if they were dominant in English. Thus, properties of a second language can lead to more veridical perception of certain sound sequences. Similarly, properties of a speaker's L1 can confer an advantage in L2 speech perception, compared to native speakers, as seen in a study by Chang and Mishler (2012) on Korean-English bilinguals' perception of word-final unreleased stops in English. Word-final stops are obligatorily unreleased in Korean, but optionally so in English, which Chang and Mishler linked to a measurable advantage in perceiving the place of articulation in the absence of a stop release.

Considering these empirical issues together, an ideal architecture must account for the gradient nature of knowledge in the form of mental representations, which is sensitive to the possible overlap of grammatical information from two or more (competing) source grammars. These mental representations consist of multiple levels of linguistic information, which leads to the potential of both vertical and horizontal overlap and conflict. In addition to establishing and declaring the (typological) (dis)similarities of both source grammars, this architecture must also establish equivalence amongst categories and constraints in the conjoined “common grammar.” In summary, and agreeing with Cook (2016, p. 18) once again, “the mental representation of language is a complex system with all sorts of internal and external relationships; it may be quite arbitrary to divide a bilingual system into separate areas, modules, and subsystems, that can be called languages in the plural.”

The variable, dynamic nature of these mental representations is the result of an architecture that embraces the fact that competition amongst these factors is the norm rather than the exception. The final mental representations are thus conditioned and shaped by both internal and external factors that operate perhaps on different time frames and exhibit unique developmental histories. Such is the nature of a dynamic system, whose core attributes are listed by de Bot (2016, pp. 126–30):

- Sensitive dependence on initial conditions
- Complete interconnectedness
- Non-linearity in development
- Change and development through internal reorganization and interaction with the environment
- Systems are constantly changing
- Dependence on internal and external resources
- Systems may show chaotic variation over time
- Development is conceived of as an iterative process

According to the integrationist perspective taken here, in addition to the gradient nature of knowledge in the form of mental representations we also adopt these conditions. Importantly, as explained by MacWhinney (2005, p. 191), “What binds all of these systems together is the fact that they must all mesh in the current moment. One simple view of the process of meshing is that cues combine in an additive manner (Massaro, 1987) and that systems are partially decomposable (Simon, 1969).” An attractive outcome of viewing the language development, maintenance, and activation/usage of bilinguals as a dynamic system is that it stands to bring generative models more in line with emergent (Kirby, 1999) and Bayesian (Culbertson, 2010) approaches to the development of grammar systems.

The shift toward a dynamic system with gradient representations raises questions concerning the compatibility that such a model might share with currently existing frameworks. Again, here we seek to outline how much these current frameworks can handle these important architectural adjustments. In our view, representations that are “partially decomposable” (Simon, 1969) are best interpreted as distributed knowledge that combines to deliver complex representations. There are multiple ways to postulate how these complex representations come into existence, from the use of declarative and violable constraints (van Oostendorp et al., 2016; Putnam, 2017) to those that employ an architecture of grammar with an invariant computational syntax (Kandybowicz, 2009; Lohndal, 2013; Boeckx, 2014, 2016; Grimstad et al., 2014; Alexiadou et al., 2015; Riksem, 2017). Questions regarding the difficulty in arriving at the proper definitive set of universal parameters and the inability to determine if and how these constraints could combine to deliver complex representations had emerged in the work of Newmeyer (2004, 2005) and has led to a reappraisal of the role of the traditional notion of parameters (see e.g., Fábregas et al., 2015; Eguren et al., 2016). What the majority of these recent proposals have in common is the move from parameters to features and cues that are either distributed across multiple levels or realized as associations that are united with a particular combination of derivational units (as is the case in Distributed Morphology, DM). The result from this exploration is that these mental representations consist of multiple levels and simultaneously display complex and atomic natures (cf. Quine, 1940). Under such assumptions, both lexical items (= lexicon) as well as more complex units (= syntaticon) (a la Emonds, 2000) are generated in similar fashion. Gallego (2016, p. 157) suggests that such an approach, i.e., one where items in a lexicon and syntacticon (i.e., the storage of fused units—chunks—typically larger than a lexical item) exhibit a dual atom-complex nature, must address the following questions:

Q1 : What is the set of morphosyntactic features {F} that UG provides?
Q2 : How do these features bundle to form LIs (= lexical items)?
Q3 : Why is LI-internal structure opaque to computation?

Gallego (2016, pp. 157–158) advances a system,

*where syntax recycles complex sound-meaning pairings as brand new units of computation, creating a loop between syntax and the lexicon (roughly as in Starke 2010). A way to conceive of this relation would be the warp the standard “Y Model” into what we would call a “U-Turn Model” collapsing the pre-syntactic lexicon and the post-syntactic interpretive components into a unique interface that would communicate with other cognitive modules (the C-I and S-M systems)*.

The proposal of such a U-Turn Model enables communication between coexistent systems that is “highly reminiscent of the syntagmatic-paradigmatic distinction” (Gallego, 2016, p. 158). Furthermore, such an architecture is similar in scope and design to other proposals in the literature such as those put forward by Uriagereka (2008) and Stroik and Putnam (2013). As pointed out by Stroik and Putnam, such an architecture supposes that the Faculty of Human Language is situated *within* the performance system, which resonates with connectionist models of cognitive processes and their neurobiological implementation. Neural networks consist of interwoven neural links that “criss-cross in a three-dimensional curved grid structure” and “this grid structure contains highly ordered neural overlaps” (Stroik and Putnam, 2013, pp. 14–15; see also Ramachandran, 2011; Weeden et al., 2012). We maintain that in this overlapped architecture, the common grammar shares a unified semantic representation, which connected with other levels of linguistic information (i.e., phonology, morphology, and syntax). Two additional points are in order here as well: First, appeal to a U-turn-type architecture does not implicate that (strictly) modular models are preferred over parallel ones. On the contrary, as discussed by van Oostendorp et al. (2016), a parallel architecture with limited degrees of overlap among the sub-domains of grammatical knowledge is fully capable of deriving modularity. Second, O'Donnell (2015) shows that not only is the overlap (to some degree) expected between domains of grammatical knowledge, but also that the notions of storage and computation should not be viewed as completely separate entities. In fact, the notion of *fragmented grammars* that he advances in his treatment of probabilistic parsing shares significant overlap with the integrated approach we develop here. We can translate this idea to other representations of grammar as well: a system involving some ranked “soft” constraints that are violable can explain empirical data showing when and why judgments of acceptability are graded (Keller, 2000; Haegeman et al., 2014)^6^.

This brings us once again to the notion of cross-linguistic proximity and the challenge of capturing and measuring this heuristic without the aid of traditional parameters. The move away from traditional parameters toward e(xternal)-parameters raises interesting challenges for the ontology of a model (see e.g., Putnam, 2017; Putnam et al., 2017 for an overview). The challenge for finding proximity and congruence between two source grammars requires a multi-dimensional search. This situation is once again a bit more complex in the bilingual mind, where the separation of individual source grammars is essentially not possible. The notion of proximal and distal has been a cornerstone in research on cross-linguistic influence (CLI) in sequential L2 acquisition. For example, Kellerman (1995, p. 125) states:

At its simplest, the L1 can be seen as a direct cause of erroneous performance, especially where such performance is shown to vary systematically among learners with different L1 backgrounds…

The concept of “error” is difficult to define in the context of L2 (and in bilingual language production *a priori*), given that other factors such as language mode, cognitive load, and other extraneous factors can impact linguistic output. Once again, one of the primary culprits in this line of thinking is “monolingual bias,” that (i) “there are separate language systems that have an impact on each other,” and (ii) “languages exist as stable entities in our brain” (de Bot, 2016, p. 133). The very existence of joint representations (i.e., of competence) also address this problem, which reduces notions of language contact since Weinreich (1953) to (non-)facilitative “transfer” as a fluid continuum of bidirectional influence (e.g., Schmid and Köpke, 2007; Seton and Schmid, 2016).

What is more, it is unclear at present how much of an aid or a hindrance similar linguistic information can be. McManus (2015) shows that the high degree of overlap between the aspectual and tense systems in English and French can pose a challenge for the acquisition of French an L2, and the notion that substantial but incomplete overlap presents greater challenges to learners than more distant correspondences has long been current in the literature on bilingual phonetics (Flege, 2003; Best and Tyler, 2007). With respect to research on code-switching, establishing congruence across categories appears to be essential in hybrid outputs (Deuchar, 2005). To date, it is unclear what role typological proximity may play in L1 attrition, although current research hopes to provide some insight into this matter (Schwarz, in progress). In the next section, we take on the task of providing a detailed overview of the fundamental components of our model.

## Model: core components

We put forward a dynamic model of linguistic representations that shares representations between languages that change over time in response to experience. We note that the encoding strategy in our model is not specific to language. Rather, it is an instantiation of general cognitive mechanisms that encode either declarative knowledge or procedural programs. As a consequence, proximity between languages is determined by how frequently shared representations are used in processing each language. We contrast the concept of this inherent proximity from such proximity that is the result of a cultural-evolutionary process, which, of course, has resulted in more or less co-representation between any two given languages.

Typological distance is a result of divergence in a vector space that is spanned by the representations that define syntactic operations in each language. *Compression* describes how shared representations form between those grammar representations associated with each language. In cognitive psychology, *chunking* is an example of such a compression operation. Similar to chunking, we assume that these grammatical representations are built up empirically; yet, they are probabilistic (symbolic and subsymbolic), and they dynamically change as language is used. Compression facilitates the efficient encoding of constructions in each language, with a construction being represented by a vector in space. The *cosine* metric is a common method to characterize the distance (angle) between two vectors in representational space. Then, the mean distance between constructions situated in this shared vector space describes typological similarity. In other words, if languages are represented in similar areas of this space, they are deemed similar, and facilitate and interfere with each other. If languages end up in more distinct clusters, they are typologically more distant, and may interact to a lesser degree.

With this model, we embed linguistic representations in a more general program of distributed (semantic) representations that have been empirically successful in describing human memory, in a psychological sense (Landauer and Dumais, 1997; Jones and Mewhort, 2007) but also language, in an engineering context (Mikolov et al., 2013). All of these approaches define some way to compress representations—often, from an initial vector space with several hundred thousand dimensions into a vector space with, e.g., 300 dimensions. This compression, also called *dimensionality reduction*, achieves generalization of the acquired representations while preserving much of their distinctiveness.

In the following, we discuss four candidate algorithms for compression that can form part of the model. Not all of them make different predictions, but they represent different cognitive mechanisms that have different neuropsychological correlates. All of them share the idea of *compression*, in that they make storage of representations more efficient over space, and/or over access time, and all four could result in a loss of information (i.e., all are *lossy*).

### Chunking

In light of limited memory resources, humans apply an effective technique to recognize commonly used combinations or sequences of signals, storing them as a single, declarative memory item. For example, the sequence ***BBCPHDCIA*** might be stored not as nine letters (exceeding most people's working memory capacity), but as three well-known acronyms, becoming an easily storable three-item sequence. Chunking has been found at many levels, from perceptual/sensory information to high-level reasoning. Efficient memory encoding, using chunking strategies, has been shown to be a hallmark of expertise (a classic of cognitive psychology: Chase and Simon, 1973). Chunks may capture lexicalized sequences of words, or they may bind related ideas. It bears repeating, that an appeal to chunking does not necessarily come at the exclusion of a minimalist model of syntax/computation. As pointed out by Adger (2013, p.c.), the idea that the initial stages of language acquisition begin with a limited, yet invariant narrow syntax and then eventually move toward a system of chunked representations is not inconsistent with some versions of minimalist theorizing. Whether this assertion can be upheld is beyond the central claim of this paper; however, we would like to point out that the decomposition of these complex units (i.e., chunks) in order to determine the degree of typological similarity requires a compression-unpacking algorithm discussed here (see e.g., Christiansen and Chater, 2016).

### Routinization

Chunking has its equivalent in learning procedures and sequences. A repeatedly successful sequence of cognitive operations may be combined into a larger one (Anderson, 2013). This principle may apply to goal-oriented actions in the same manner as to linguistic phrases, so that syntax can be represented as a system of routines (Jackendoff, 2002). Within a parallel architecture of grammar, distributed units of information (call them *features*) can become routinized within particular levels of grammars as well as in combination with others (with the aid of functional mapping). As particular combinations of language (on multiple levels) are more frequently used/activated, the units as a whole become easier to generate and comprehend, thus facilitating efficiency as well as reducing entropy in the prediction of immediately preceding units. These units become highly routinized. One interesting consequence of this interpretation of the generation and routinization of linguistic chunks is that it blurs the clear distinction between elements that are exclusively regarded as stored elements of declarative knowledge vs. those that are generated as the result of computational operations (cf. O'Donnell's (2015) notion of *fragmented grammars*).

### Distributed representations and declarative memory

In a distributed model meaning is represented as a composition of weighted references to other meanings, to episodic experiences, or in arbitrary feature space. The *distributional hypothesis* states that words that appear in the same context share (some) meaning. So, we begin with a feature space that is composed of as many dimensions as there are contexts (practically, documents or paragraphs in text). Then, each word is represented as a vector of binary values that describes which contexts the word occurs in, defining the meaning of a word in terms of its usage. Consequently, similar meanings are then represented in nearby locations, or *embeddings*, in this vector space.

The semantic space is optimized in order to maintain a unique representation of meanings while simultaneously ensuring computational efficiency. Throughout language use, it can also be gradually optimized to improve understanding or producing language in context by using predictions about related meanings. Earlier forms of vector space models, such as *Latent Semantic Analysis* (Deerwester et al., 1990), apply a mathematical operation that reduces the dimensionality of such spaces systematically.

Modern architectures (e.g., Mikolov et al., 2013) are optimized to actually predict a word given its context, i.e., its left and right neighbors. Thus, while these representations can capture some local syntactic regularities, they are not designed to represent syntax more generally. However, it is easy to see that the algorithm that reduces dimensionality and thus determines the encoding is a form of compression: It allows for a more efficient representation of meaning. The representational principles associated with distributed semantic encoding are not limited to the domain of semantics. Syntactic knowledge has rich stochastic ties to semantic representations, and can be seen as configural constraints that display a mix of regularities and exceptions. Distributed representations may well be a neurologically and psychologically plausible framework for syntactic knowledge, and it is a technically realistic candidate (Kelly et al., 2013, 2017). At lexical, syntactic, and morphological levels, the overlap in semantic space and joint compressibility of lexicons associated with two languages determine their mutual facilitation. As we discuss below in the section Testing Our Model, we suggest that these levels exist in parallel with their semantic counterparts occupying another layer of parallel structure (i.e., there exists only one shared semantic/conceptual structure).

**Figure 1:**
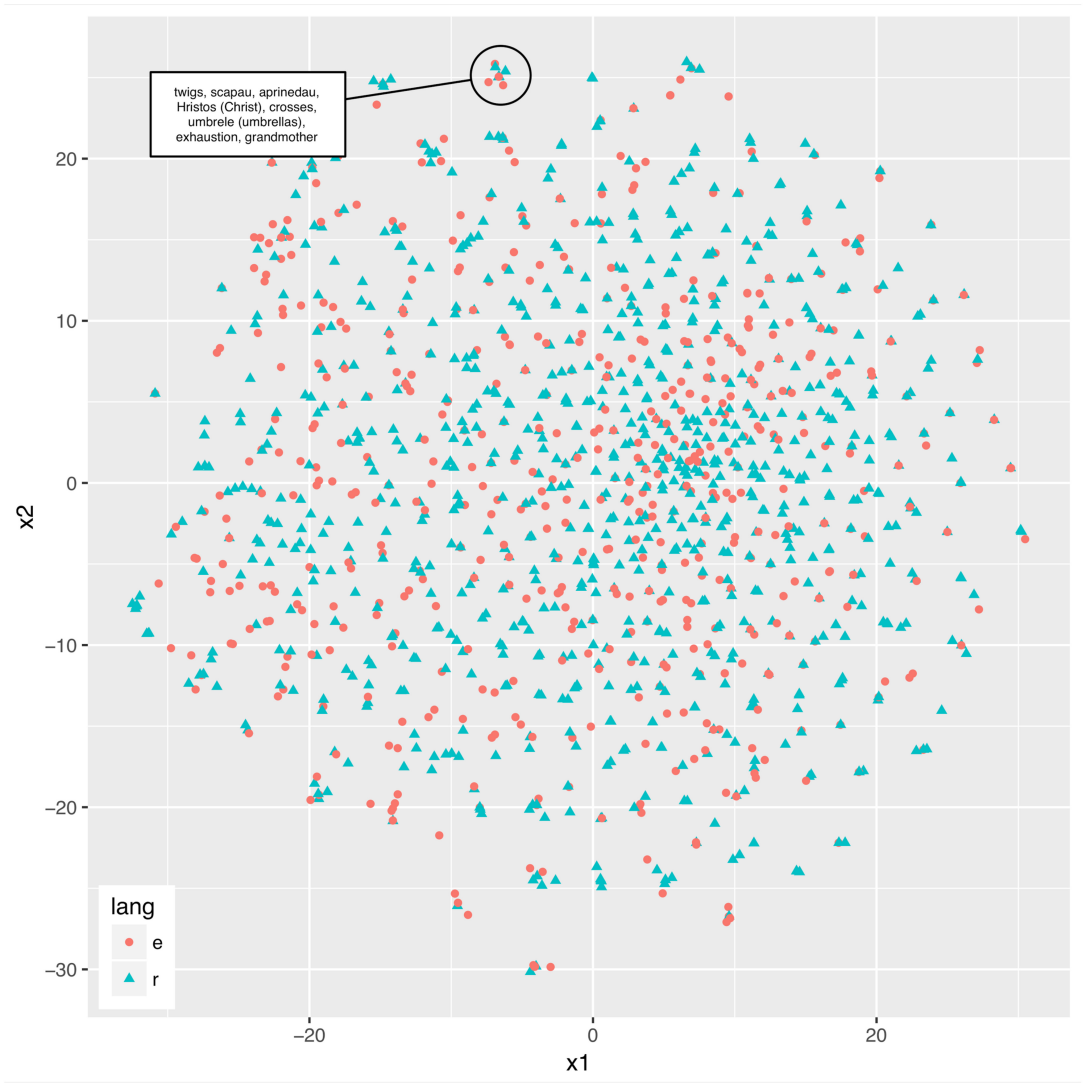
Two languages sharing the same lexical-semantic space. Distributed semantic representations for 1,500 word samples were acquired from a parallel Romanian (“r”) and English (“e”) newspaper corpus and reduced to a two-dimensional vector space using T-SNE for demonstration purposes.

We provide an example of joint representation in compressed vector spaces in Figure 1. Here, a 1-million-word corpus of parallel Romanian and English newspaper texts was used (Mihalcea and Pedersen, 2003). A semantic space was obtained from a term-document matrix (sample of 1,500 words, 1,050 documents per language), which associates each term with the documents (or paragraphs) it occurs in, and their frequencies. This space, thus, characterizes word meanings in terms of their co-occurrences. A recent, high-performing dimensionality reduction technique that has a neural implementation was used (t-Distributed Stochastic Neighbor Embedding, van der Maaten and Hinton, 2008) to produce a two-dimensional vector space shared by the two languages. Figure 1 shows the words and their locations in space. Note that a plausible model of such representations will use on the order of 300 dimensions rather than two, and it will result in regions of shared and regions of language-separate semantic-syntactic representations. Such a model would resemble models such as Kantola and van Gompel (2011) and de Bot (1992), in which related representations are connected (such that they can influence each other in processing), and unrelated representations are not, but in our model, the connected representations would be shared, as in Hartsuiker et al. (2016). However, the multi-dimensionality of the representational space would permit sharedness to be gradient. Although for the immediate purposes of illustration Figure 1 only provides a two-dimensional representation, there is clear room for expansion of vector space that could include other level of grammatical information (e.g., morphology and syntax).

### Compression algorithms from computer science

Compression has its equivalent in computer science. In lossless compression, arbitrary but not random sequences can be represented by replacing frequent subsequences with references to an *ad hoc* table (Ziv and Lempel, 1978). A commonly used example of this principle (in an improved version) would be the popular *Zip* program. Lossy compression allows for differences between the target and the representation retrieved from memory, which is typically used where psychophysics or cognitive phenomena will prevent humans from perceiving the differences, such as in sound (e.g., *MP3*) or vision (e.g., *JPEG*). In terms of grammatical representations, our proposed model posits that compression of sequences of linguistic representations or of grammatical knowledge is a representation of encoding of grammar, including that shared between languages.

Grammatical representations start with declarative representations, which are routinized as a result of their use (see, e.g., Reitter et al., 2011 for such model, and Anderson, 2013 for a cognitive architecture that describes this routinization process). Repeated use of a sequence of memory retrievals leads to their compilation into fast routines that do not require memory retrievals. This process combines short subsequences first, and throughout repeated use, the resulting new chunks are combined again. This mirrors commonly used compression algorithms (Ziv and Lempel, 1978).

Grammatical encoding in the model depends on distributed representations to account for semantics, and it is possible that distributed (neural) representations can account for (some) syntactic knowledge as well. We propose an account of compression that combines this symbol-level compression and the notion of compression of representational spaces. At first sight, compression through routinization (psychology) or lookup tables (computer science) would be applicable to representations of syntactic procedures, while compression of semantic spaces would apply to distributed semantic representations. We propose that semantic and syntactic spaces are represented jointly. Compression at the symbolic level, akin to chunking, is lossless, while compression of representational spaces is lossy.

The architecture we discuss is compatible with accounts of “Shared Syntax,” and with empirical data that shows cross-linguistic priming (e.g., English-Spanish, Hartsuiker et al., 2004), as noted in the previous section. An instantiation of the architecture will need to explicate how syntax is represented and how it is compressed; this would yield predictions for the facilitatory and inhibitory effects of an L2 on an L1 in language performance, a point which we turn to in the following section.

## Testing our model

Admittedly, the programmatic model put forward here does not yet constitute a fully implemented model. The principles on which our proposal is founded nonetheless make testable predictions. For example, there is debate over whether structural priming effects are best accounted for through models in which bilinguals' syntactic representations are shared vs. separate (Bernolet et al., 2007; Kantola and van Gompel, 2011; Hartsuiker et al., 2016). At first blush, our model shares a salient affinity with Hartsuiker et al.'s shared syntax model, but by integrating two grammars through lossy compression, we actually bring together both the separateness and sharedness of syntactic representations. We thereby aim to reconcile apparently conflicting findings by predicting when results will support a shared vs. separate (but interacting) syntax model.

As a second example, representational similarity as predicted by the account of distributed representations (and possibly more symbolic chunking) leads to observable behavior, such as facilitation of jointly represented constructions through syntactic priming and the difficulties encountered when attempting to rapidly compress grammatical information where typologically-contrastive information is present. As a point of illustration, consider the following hybrid representation reported by Karabag (1995) (cited by Treffers-Daller, 2017; German appears in regular font, Turkish in *italics*, doubled elements are underlined):

(3) Deutschland muß mit dies-en Hippie-*ler*-*le* *ba?-a*
Germany must mit this-dat Hippie-pl-instr. head-dat
ç*ik-ma-si* *gerek**-iyor*.
leave-nom-3sg must-Pr.Prog-Ø
‘Germany must cope with these hippies.’

In the example above (3), there are two instances of doubling, one involving the doubling of modal verbs (G: *muß*/T: *gerek*) and two adposition elements (G: *mit*/T: *le*). Congruence is established in the common grammar with respect to dative/instrumental case. Although German does not license independent morphosyntactic forms of instrumental case, it is subsumed as a sub-function of dative case in this language. In this structure, the lexical item *Hippie* is double-marked with dative/instrumental case, which we predict is likely due to the difficulty encountered in the common grammar to rapidly compress structural information (i.e., syntax) when one of the source grammars is a fusion-language (German) and the other an agglutinating-one (Turkish). To further illustrate this point, we provide a sketch of a formal analysis of this structure in (4) below making use of the Simpler Syntax framework (Culicover and Jackendoff, 2005).

(4) Phonology: /mit dies-en Hippie-*ler-le ba?-a çik-ma-si gerek-iyor* /
Morphology: [G: Dative/intrumental_*dies-en* / T: N-*le*]
Syntax: [_VP1_ V(modal) [Det-N(plural) V(leave)] V(modal) _VP2_
Semantics: [Progressive(COPE([INSTRUMENTAL/ DATIVE(with) [Hippies; DEF]]]))

Working from the bottom up, we see unified semantic content; i.e., “X must cope with these hippies” in this representation. With respect to the syntax, we propose two separate verb phrases (VP1 and VP2, respectively) that overlap where the modal verbs from each respective language appear at the edge of each VP based on the preference associated between the source grammar home of the verb and the VO- vs. OV-preference^7^. The lexical verb *gerek* “to leave” serves as the anchor of these overlapping VPs (see e.g., Chan, 2008, 2009, 2015; Goldrick et al., 2016a for similar arguments). We propose that there is an additional level of structural (i.e., syntactic) overlap with respect to the determiner/noun phrase (D/NP), with the noun *Hippie* serving as the anchor. Morphological marking indicating the dative/instrumental case on this noun appears both on an independent determiner (G: *dies-en* “these”) and as an agglutinating morpheme (T: *-le*). Crucially, the appearance of such hybrid units are dependent on some degree of congruence (i.e., the semantic representation and the recognition that dative case in German and Turkish instrumental case are approximate equivalents) as well as some degree of typological divergence (i.e., the fact that German is typologically classified as a fusion-language, whereas Turkish is agglutinating) in the source grammars contributing to the integrated, common grammar. Admittedly, models based on separate representations for each language could also account for data such as these, but we argue that an integrated model does so more naturally and, more importantly, it can do so in a way consistent with how bilinguals produce and process such structures in real time.

A third and final example of the role of typological relatedness in determining the possibility of doubled elements in the outputs of bilinguals comes from Austin's (2015, 2017) research on the acquisition of Differential Object Marking (DOM) and pre-verbal complementizers in the speech of young, bilingual Basque-speaking children in contact with Spanish. These two particular languages contrast in significant ways, with Euskara, the Basque language, marking DOM-effects with a dative verbal suffix ***zu***-, and Spanish realizing DOM-effects by means of a preverbal marking ***a*** [compare (5a) and (5b) below; both from Austin, 2015]:

(5a) Basque DOM^8^
*Nik zuri entzun di*- ***zu***- *t*
Erg1sg Dat2sg hear Abs3sg- Dat2sg- Erg1sg
‘I have heard you-Dat.’

(5b) Spanish DOM
*He visto* ^*^(***a***) *mi hija*.
Have-1sg seem DOM my daughter
‘I have seen my daughter.’

A second structural trait that distinguishes Basque and Spanish concerns the order of constituents in a clause; i.e., Basque is head-final language, whereas Spanish adheres to a head-initial ordering of constituents [compare (6a) and (6b); both from Austin, 2015]:

(6a) Basque (head-final)
Guk liburu asko irakurri dugu
We-Erg book a lot read Aux-Abs3sg-Erg1sg
‘We have read a lot of books.’

(6b) Spanish (head-initial)
Nosotros hemos leí*do* muchos libros
We-Nom have-Nom1sg read many books
‘We have read many books.’

One of Austin's key research questions focused on the appearance (or lack thereof) of preverbal complementizers in the speech of monolingual and bilingual Basque-speaking (and –acquiring) adults and children. To broadly summarize her findings, Austin's study revealed the somewhat unexpected result that monolingual children produce more instances of DOM than bilingual children. With respect to the use of preverbal complementizers, Austin (2015, p. 10) provides the following rationale for her findings:

*The use of pre-verbal complementizers presents a very different developmental pattern. These forms are used exclusively by four bilingual children*^9^ *between the ages of 2;08 and 3;02, and were never produced by monolingual children or adults. Five bilingual children in this age range never used them at all, and their production does not seem to be correlated with their MLU in Basque. […] I understand these utterances as a temporary relief strategy which may be used by some bilingual children when they are confronted with a construction that they have not yet acquired, following proposals by Gawlitzek-Maiwald and Rosemary (**1996**) and Bernardini and Schlyter (**2004**)*.

Example (7) below (from Austin, 2015) illustrates this non-target structure, where a non-target preverbal complementizer appears:

(7) *zergatik badoa eskuelara*
why-Comp go-Abs.3sg school-to
‘Because s/he goes to school.’

Interpreting Austin's findings through the lens of the integrated model of bilingual language and cognition that we adopt here, this relief strategy may likely be the result of elements from both source grammars simultaneously competing for a finite space of representation in syntactic structure. Under such conditions of typological contrast, the elements from both grammars with occasionally appear together, resulting in hybrid code-mixing. Of particular interest, these structures are only found in developing bilingual grammars, and crucially not in the speech of adults or monolingual children^10^. In summary, Austin's findings and suggested explanations are largely consistent with both ours and Muysken's (2013), where the linguistic output of bilinguals is the result of a (complex) optimization process.

As we have discussed throughout this article, grammatical knowledge—especially in the case of the bilingual mind—is best understood as a multi-dimensional, multi-vector space. In order to combat the need to produce and comprehend grammatical information with a high degree of efficiency, compression is applied whenever possible. One potential strategy to avoid the loss of (important) information, due in no small part to the lossy information within symbolic chunks, is to represent structural information twice. Although hybrid representations are commonly found in both typologically similar and dissimilar languages (see e.g., Braunmüller, 2009 and his work on code-switching among Danish-German and Danish-Faroese bilinguals), we suggest the presence of doubled elements in both developmental grammars and in simultaneous code-switching data represent solid evidence in favor of the dual activation of elements from both source grammars. Here we make the prediction that the difficulty to compress linguistic information into a common bilingual grammar consisting of source grammars that differ on at least one level of linguistic information, will lead to a higher degree of doubled structures. A preliminary survey of the nascent literature on doubled-elements in code-switches supports our hypothesis (see e.g., Chan, 2009, 2015; Goldrick et al., 2016a, and references therein). In contrast, we anticipate that doubled elements in hybrid representations will be far less likely in outputs when the source grammars exhibit higher degrees of (near) typological overlap. For example, we predict that it would be less probable to find doubled adpositions where both source grammars license prepositions [i.e., English-German ^*^mit with der Seife (with-G with-E the-dat soap)]^11^. What is yet to be determined is how much overlap across which particular levels of grammatical information represents important thresholds for any particular increase in the appearance of such forms; however, such hypotheses are indeed testable through the analysis of existing corpus data as well as experimental research with code-switching populations.

Finally, by implementing the compression algorithm in cognitively plausible ways (Anderson, 2013), our model aims to explain the various phenomena associated with bilingualism, as well as second language learning as grounded in the ways that general cognitive mechanisms interact with linguistic experience. With regard to second language acquisition in particular, a compression algorithm makes specific predictions about how learners perform the integration of new and existing linguistic knowledge, including differences based on specific language pairs or individual differences between learners.

## Conclusion

Although sufficient evidence exists supporting the importance of typological similarity and distance in the acquisition, spontaneous speech, and attrition of bilingual grammars across the lifespan, deriving a working definition of this concept with predictive power has been a challenge in both generative and cognitive models of language. Here we make the case for a multi-dimensional, multi-vector network and a hybrid symbolic/sub-symbolic cognitive framework, which we deem to be necessary to model linguistic representations. In our view, this approach leads to a more accurate view of typological relatedness in both stored/routinized elements (i.e., lexical items and larger chunks) and their interaction with one another. Modeling bilingual grammar through the lens of this architecture, as we propose here, enables us to establish a depiction of the reality of dueling grammars and the routinization, chunking, and compression operations that take place in establishing congruence among elements of these grammars. There are many diagnostic tools that allow us to evaluate such a model, including code-switching phenomena, typological relatedness as evidenced by facilitation of L2 acquisition, or resistance to attrition. The model put forward here can be extended and adapted into various models, such as, but certainly not limited to, exo-skeletal frameworks of grammar where traditional parameters have been externalized from any sort of universal, or narrow computational system.

## Author contributions

MP was the lead author for this manuscript, developing the outline and core ideas for this manuscript. DR contributed to the overall editing and added valuable insights from cognitive science and computational applications of language science. He was the primary author of section Model: Core Components. MC contributed throughout the paper to strengthening the overall argumentation of the manuscript.

### Conflict of interest statement

The authors declare that the research was conducted in the absence of any commercial or financial relationships that could be construed as a potential conflict of interest.

## Footnotes

^1^As a point of clarification, our definition of hybrid representations is a cover term for linguistic outputs that contain elements from multiple source grammars (e.g., lexical items, morphological units, syntactic elements, etc.). This is not to be confused with more narrow definitions of hybridity provided by scholars such as Aboh (2015), which applies directly to creoles and mixed languages. Although we do not directly discuss how these grammars would fit into our model due to space considerations, it does appear that this would not pose significant difficulties.

^2^As pointed out by a Frontiers reviewer, there is also ongoing work to refine the notion of parameter in generative theorizing (e.g., Baker, 2001, 2009; Fábregas et al., 2015; Eguren et al., 2016; Biberauer and Roberts, 2017). Two particular recalcitrant issues concerning the notion of parameter that are of relevance to our model are: (1) the grain-size of parameters (e.g., Westergaard, 2013), and (2) the vertical and horizontal interaction of different sets of parameters (e.g., Biberauer and Roberts, 2017; Putnam, 2017). Here we adopt an agnostic approach to the notion of parameter, recognizing that in a multi-dimensional grammar space capturing the interaction and competition of these units is of primary importance.

^3^Although the concept of lossy representations is commonly associated with constructionist and usage-based approaches (e.g., Goldberg, Forthcoming; Lau et al., 2016), it has also played a role in shaping generative approaches (e.g., Featherston, 2005, 2007; Pater, 2009; Goldrick et al., 2016a,b; Putnam and Klosinski, 2017).

^4^An interesting point about two languages in the mind of bilinguals is that they may merge into a unified system at different time scales. It may occur in the mind of an individual who is becoming bilingual (whether simultaneous or not), but it also occurs at the level of a bi- or multilingual speech community. Something like this reasoning already appears in research on pidgins and creoles, but what we discuss here is broader than that, and could apply to any situation where there is language contact.

^5^Technically speaking, in the model we propose here, there is no distinction between blends and mixes as suggested by Putnam and Klosinski (2017). From the perspective of a multi-dimensional architecture, the difference between the two examples above reduces to the number of levels where the two source grammars do (not) overlap.

^6^A Frontiers reviewer raises the question as to whether or not there are fundamental differences between the “soft” constraints that we mention here versus those found in Optimality Theory. Although they share the trait that they are violable in nature, the constraints we refer to above can combine with one another, resulting in additive gang-up effects as argued for in Harmonic Grammar (Pater, 2009).

^7^We thank Ray Jackendoff (p.c.) for engaging in discussion with us about this analysis.

^8^Austin (2015) notes that the Basque DOM effect in (5a) where the dative suffix appears is common in the dialect of Basque spoken in the Spanish Basque country but is not in the Basque spoken in France.

^9^Out of a total of 20 bilingual children who participated in the study.

^10^It is important to recognize here that although typological dissimilarity apparently plays a critical role here, at this point we cannot claim with any degree of certainty which linguistic and cognitive factors may be at play here. One possible candidate would be inhibitory control; if this were a dominant factor, it would indicate that these bilingual grammars have representations that are retrievable to varying degrees, but the lack of inhibitory control leads to doubled structures. Potential evidence in favor of this hypothesis come from work by Lipski (2009, 2014) on code-mixing in dysfluent (or low-proficiency) bilinguals. We leave this for future research and thank Nuria Sugarra (p.c.) for an insightful discussion with us on these matters.

^11^Upon closer inspection, Babel and Pfänder's (2014) hypothesis that typology does not play a dominant role in “copying” phenomena does not pose a serious challenge for our model. Their argument that “speakers' perceptions of differences or similarities between languages are crucial to their development and change” (p. 254) is an appeal to the importance of congruence between simultaneously active grammars. Furthermore, in their proposal they do not addressed representations that contained doubled elements.
